# Radiation-induced premature cellular senescence involved in glomerular diseases in rats

**DOI:** 10.1038/s41598-018-34893-8

**Published:** 2018-11-14

**Authors:** Sae Aratani, Masako Tagawa, Shinya Nagasaka, Yukinao Sakai, Akira Shimizu, Shuichi Tsuruoka

**Affiliations:** 1 Department of Nephrology, Graduate School of Medicine, Nippon Medical School, Tokyo, Japan; 2 Department of Analytic Human Pathology, Graduate School of Medicine, Nippon Medical School, Tokyo, Japan

## Abstract

Currently, cellular senescence has emerged as a fundamental contributor to chronic organ diseases. Radiation is one of the stress factors that induce cellular senescence. Although the kidney is known as a radiosensitive organ, whether and how radiation-induced cellular senescence is associated with kidney diseases remains unclear. In this study, we performed experiments on 7–8-week-old male rats that received a single dose of 18-Gy radiation in the unilateral kidney. The irradiated kidneys showed hallmarks of cellular senescence, including increased SA-β-gal activity, upregulation of cyclin-dependent kinase inhibitor (p53, p21, and p16), and absence of DNA proliferation marker (Ki-67). Furthermore, combined with *in-vitro* experiments, we demonstrated that radiation-induced senescent glomerular endothelial cells acquired altered gene expression, namely, senescence-associated secretory phenotype (particularly, IL-6), which might be triggered by NF-kB signaling pathway. Pathological analysis suggested severe glomerular endothelial cell injury, as evidenced by thrombotic microangiopathy, collapsing glomeruli, and reduced endothelial cell numbers. We suggested that glomerular endothelial cells were more susceptible to radiation-induced cellular senescence. In conclusion, the current study is the first to identify the important role of radiation-induced cellular senescence, mainly derived from glomerular endothelial cells, for the development of glomerular injury.

## Introduction

Recently, cellular senescence has emerged as a fundamental contributor to chronic organ diseases^1–3^, such as cardiovascular diseases^4,5^, lung fibrosis^6,7^, neurovascular diseases^8,9^, skin injury^10^, hematopoietic stem cell dysfunction^11^, and chronic kidney diseases (CKD)^3,12^. Cellular senescence, defined as irreversible cell-cycle arrest while retaining metabolic activity^13^, is a phenomenon by which cells cease to divide further. In addition to normal senescence, in which most cells undergo senescence slowly and chronologically, premature senescence can be induced in response to a variety of stress factors, such as ionizing radiation^14,15^, hyperglycemia^16^, and oxidative stress^17^.

Radiation, commonly used for treatment and diagnosis in clinical practice, is also known to induce iatrogenic complications, especially when patients are exposed to doses above a certain limit^18^. In particular, kidney is well known as a radiosensitive organ^19^. Radiation nephropathy, first documented by Domagk in 1927^20^, spans over a long term after exposure to radiation, and is characterized by proteinuria, azotemia, hypertension, and anemia^21,22^. It causes cellular damages in all components of the kidney, including the glomerulus, blood vessels, tubular epithelium, and interstitials^23^. Among them, the most striking morphological change was reported in glomerular endothelial cells, which detached from the basement membranes^24^. Radiation nephropathy is currently classified as a thrombotic microangiopathy (TMA)^25^.

Recent studies have shown that radiation-induced cellular senescence contributes to the progress of organ diseases. For example, a local radiation with a single dose of 17.5 and 20 Gy induced cellular senescence in the lungs and heart, respectively^6,26^. Another study investigated radiation-induced senescence in brain microvascular endothelial cells, which had been exposed to a single dose of 20 Gy. They demonstrated the causal relationship between radiation-induced endothelial cellular senescence and neurodegenerative diseases^8^. However, whether and how radiation-induced cellular senescence contributes to the development of CKD is yet to be clarified.

In the current study, we aimed to examine the role of radiation-induced cellular senescence in the development of kidney diseases, particularly glomerular injury. We designed a rat model of radiation nephropathy, in accordance with the previous studies^24,27^, in which a single dose of 18 Gy was irradiated on the unilateral kidney. Cellular senescence, renal failure, and glomerular changes were analyzed over the following nine months.

## Results

### Renal failure was detected at nine months post-radiation

**Figure 1:**
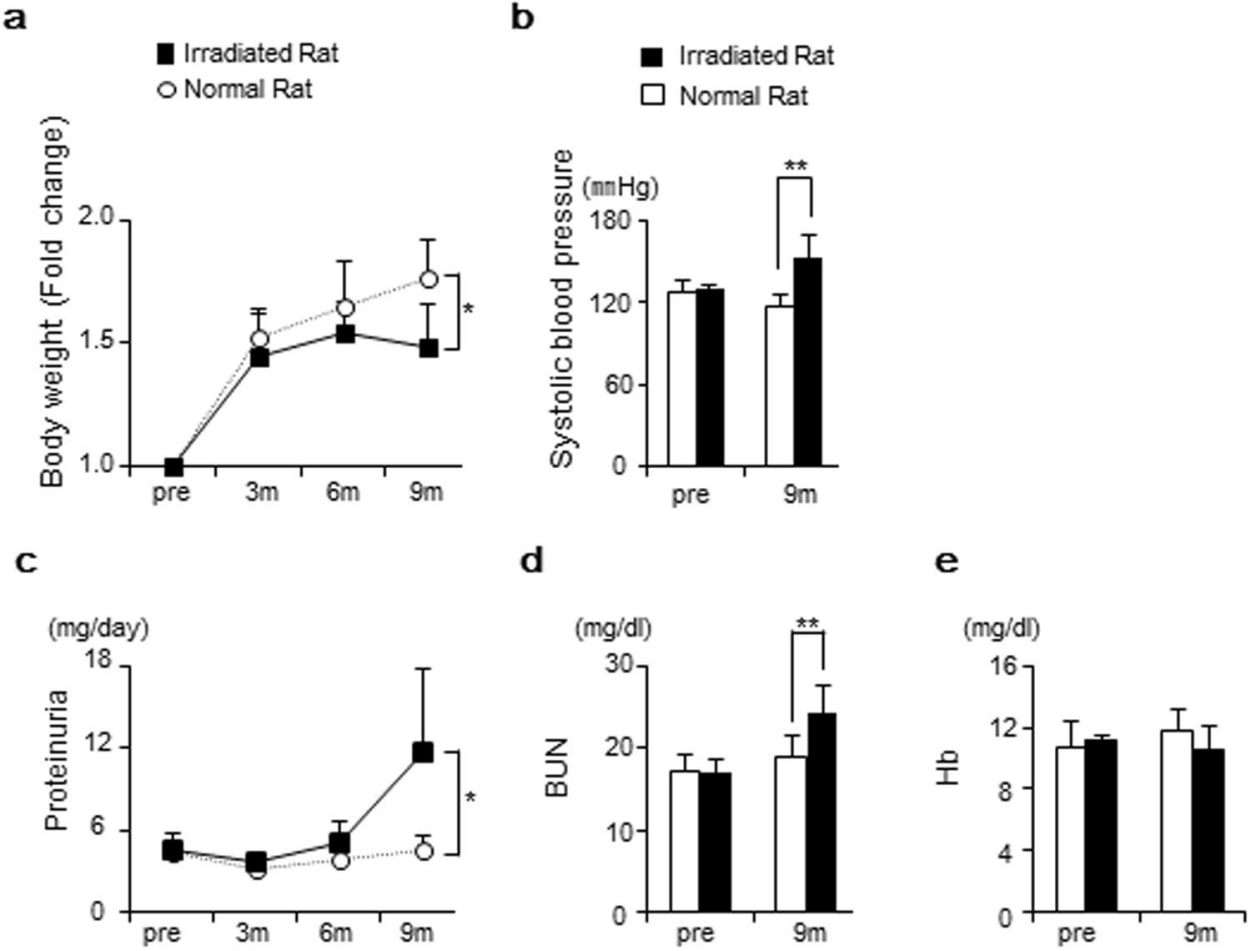
Body weight, systolic blood pressure, and renal function. (**a**) Irradiated rats showed lower increases in body weight compared to normal rats at 9 months. (**b**) Irradiated rats had higher systolic BP compared to normal rats at 9 months. (**c**–**e**) Irradiated rats showed renal dysfunction.0 at 9 months, shown as higher proteinuria and elevated BUN levels. Values are expressed as mean ± SD. *P < 0.05, **P < 0.01.

All rats included in the experiment survived to 9 months following radiation. Irradiated rats showed lower proportional increases in body weight at 9 months compared to normal rats, with means of 1.49 ± 0.17 and 1.77 ± 0.16, respectively (p = 0.021) (Fig. 1a). Irradiated rats had higher systolic BP at 9 months compared to normal rats, with means of 153 ± 16.6 mmHg and 117 ± 9.4 mmHg, respectively (p = 0.006) (Fig. 1b). Irradiated rats showed increased levels of proteinuria at 9 months compared to normal rats, with mean levels of 11.7 ± 6.1 mg/day and 4.4 ± 1.1 mg/day, respectively (p = 0.049) (Fig. 1c). BUN levels were higher in irradiated rats than in normal rats at 9 months, with mean concentrations of 24.3 ± 3.4 mg/dl and 18.9 ± 2.8 mg/dl, respectively (p = 0.020) (Fig. 1d). Irradiated rats had lower concentrations of Hb compared to normal rats at 9 months, but this difference was not statistically significant (Fig. 1e).

### Irradiated kidneys displayed thrombotic microangiopathy (TMA) and collapsing glomeruli

**Figure 2:**
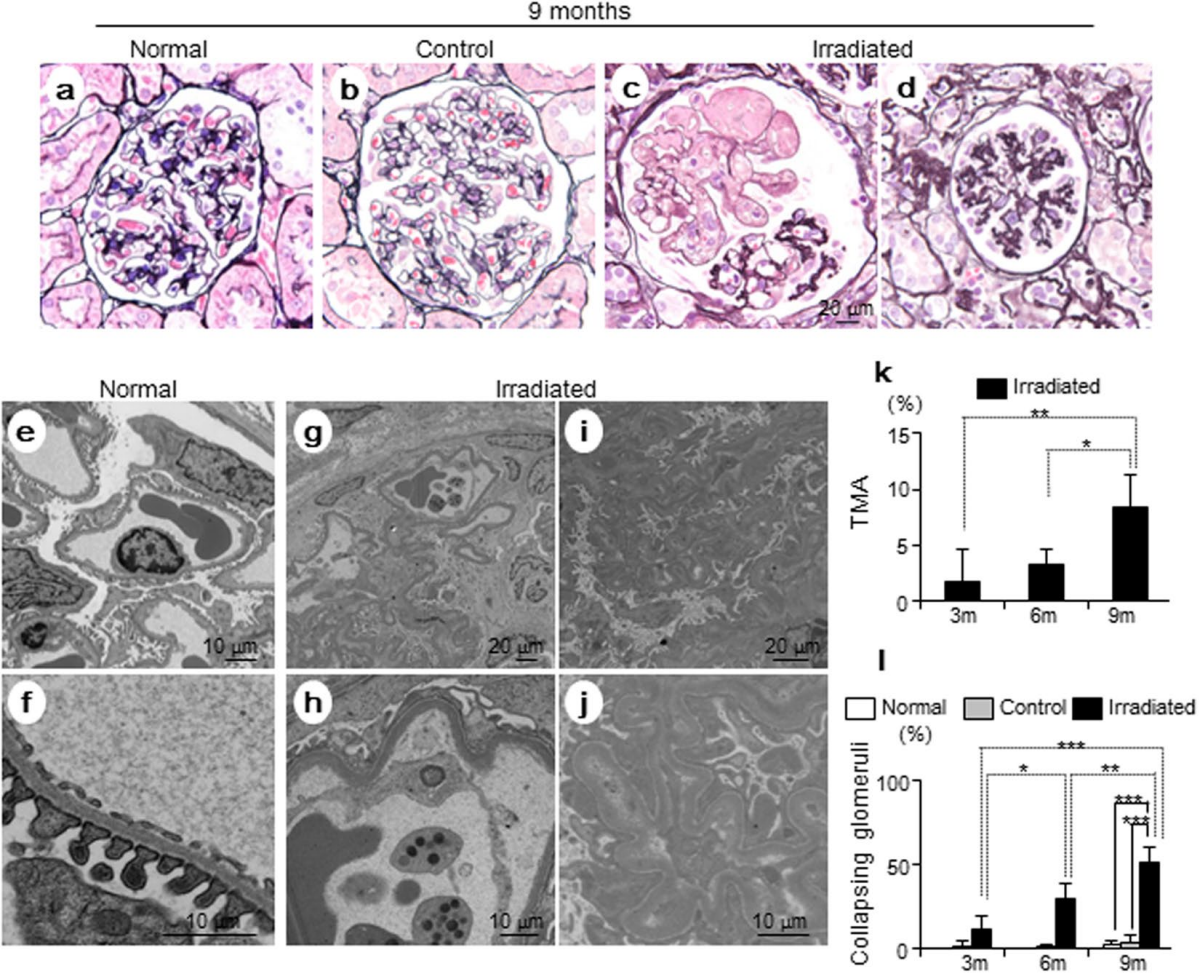
Pathological findings following radiation. (**a**–**d**, PASM stain, Bar, 20 µm) Representative image of glomeruli in normal, control, and irradiated kidneys, at nine months. (**a**,**b**) Normal and control kidneys showed preserved structure of glomeruli. (**c**) Irradiated kidneys displayed thrombotic microangiopathy (TMA) with severe glomerular endothelial cell injury. (**d**) Irradiated kidneys also displayed collapsing glomeruli with loss of glomerular capillaries. (**e**,**f**) In electron microscopic images of normal kidneys (Bar, 10 µm), glomerular capillary lumens were well preserved with fenestrated endothelial lining at nine months. In irradiated kidneys at nine months (**g**, Bar, 20 µm and **h**, Bar, 10 µm), widening of the sub-endothelial space was noted with detachment of the endothelial cells from the glomerular basement membrane (GBM) in glomeruli, indicating the development of TMA. Platelets were seen in glomerular capillaries. Podocytes were preserved with effacement of foot processes. Collapsing glomeruli (**i**, Bar, 20 µm and **j**, Bar, 10 µm) were prominent in irradiated kidneys at nine months. These glomeruli were collapsed with loss of glomerular capillaries and wrinkling of GBM. Podocytes were preserved with effacement of foot processes. (**k**) The frequency of TMA in the irradiated kidneys during the nine months post-radiation (Values are expressed as mean ± SD. *P < 0.05, **P < 0.01). TMA was present only in irradiated kidneys. The number of glomeruli with TMA lesions increased in a time-dependent manner at 3, 6, and 9 months. (**l**) Frequency of collapsing glomeruli in the irradiated kidneys during the nine months post-radiation (Values are expressed as mean ± SD. *P < 0.05, **P < 0.01, and ***P < 0.001). There was a higher prevalence of collapsing glomeruli in irradiated kidneys than in control and normal kidneys at nine months. There was no significant difference between the control and normal kidneys.

We evaluated glomerular changes using periodic acid-silver methenamine (PASM) staining and electron microscopy in normal, control, and irradiated kidneys (Fig. 2a–j). Irradiated kidneys showed severe glomerular injury, as evidenced by thrombotic microangiopathy (TMA) and collapsing glomeruli (Fig. 2c,d,g–h). TMA was only seen in irradiated kidneys (Fig. 2c,g,h,k). Notably, irradiated kidneys displayed time-dependent increases in these glomerular changes during 9 months. In irradiated kidneys, the mean percentages of TMA at 3, 6, and 9 months were 1.7 ± 2.9%, 3.3 ± 1.4%, and 8.3 ± 3.0%, respectively (Fig. 2k), and the mean percentages of collapsing glomeruli at 3, 6, and 9 months were 11.7 ± 7.6%, 30.0 ± 9.0%, and 51.8 ± 8.5%, respectively (Fig. 2l). These time-dependent increases were only seen in irradiated kidneys. Finally, at 9 months, irradiated kidneys showed higher prevalence of collapsing glomeruli compared to control and normal kidneys (irradiated, 51.8 ± 8.5% vs. control, 3.8 ± 3.7%, p < 0.001 and irradiated, 51.8 ± 8.5% vs. normal, 2.4 ± 2.6%, p < 0.001). There were no differences between control and normal kidneys at 9 months (Fig. 2l).

### Endothelial cells lessened in irradiated kidneys in a time-dependent manner

**Figure 3:**
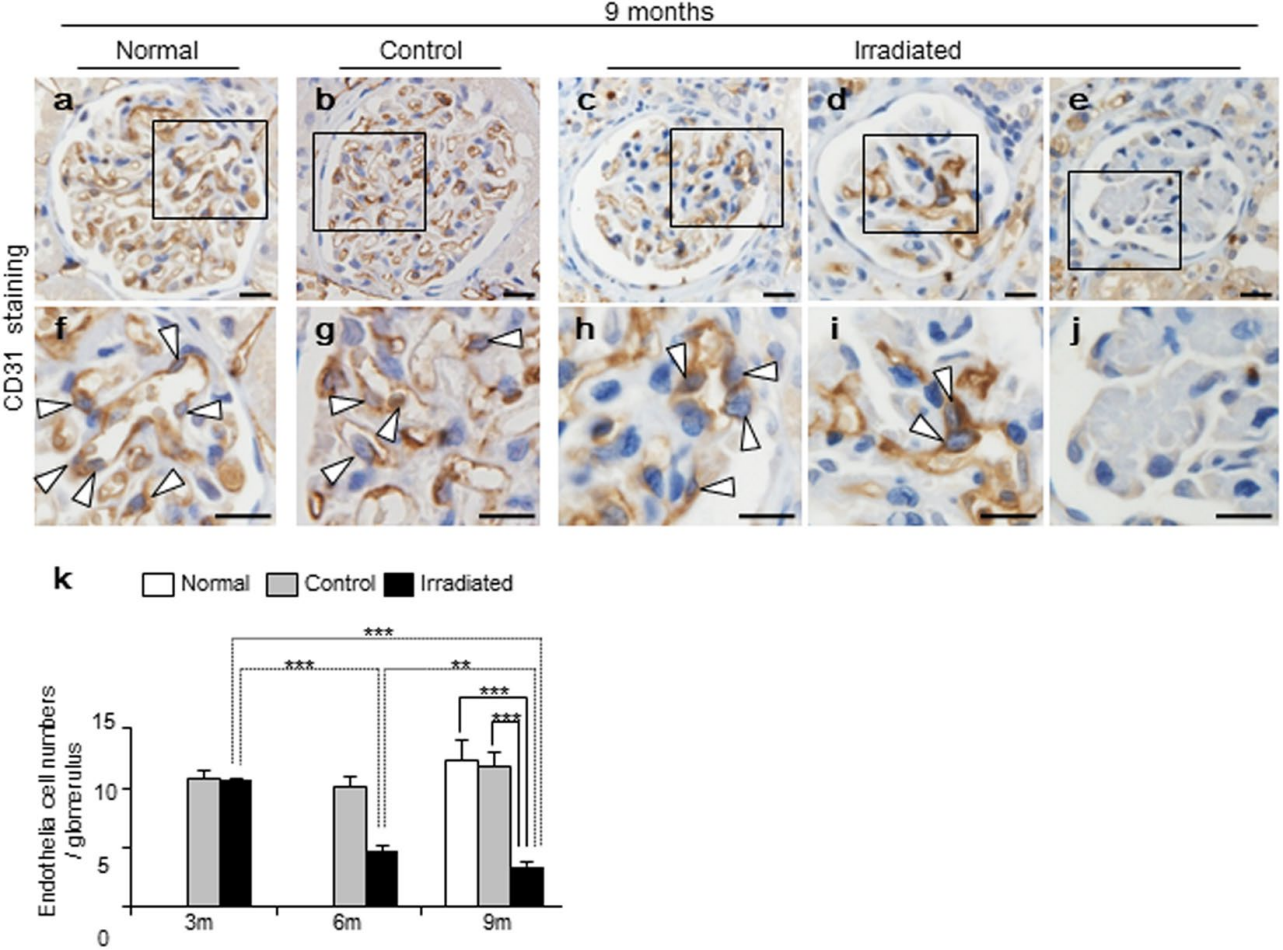
Quantification of endothelial cells by using CD31. (**a**–**j**, Bars, 20 µm) Representative images of immunohistochemistry for CD31 (brown, an endothelial cell marker), counterstained for nuclei with hematoxylin (blue). (**a**,**f**) (white arrows represent endothelial nuclei). (**c**–**e** and **h**–**j**) Irradiated kidneys showed reduced numbers of endothelial cells per tuft. (**e**,**j**) Notably, collapsing glomeruli were found to contain no endothelial cells. (**k**) Irradiated kidneys displayed a time-dependent decrease in endothelial cell numbers during 9 months. These time-dependent changes were only seen in irradiated kidneys. There were no differences between control and normal kidneys at 9 months. Values are expressed as mean ± SD. *P < 0.05 and **P < 0.01.

TMA and collapsing glomeruli suggested severe endothelial cell damage. We next evaluated the number of glomerular endothelial cells by using CD31 (a marker of endothelial cell) (Fig. 3a–j). Irradiated kidneys displayed a time-dependent decrease in endothelial cells at 3, 6, and 9 months, with mean cell numbers of 10.7 ± 0.1, 4.7 ± 0.5, and 3.4 ± 0.4 cells/glomerulus respectively (Fig. 3k). At 9 months, irradiated kidneys showed markedly decrease in endothelial cells compared to control and normal kidneys (irradiated, 3.4 ± 0.4 vs. control, 11.8 ± 1.2, p < 0.001 and irradiated, 3.4 ± 0.4 vs. normal, 12.3 ± 1.7, p < 0.001). There were no differences between control and normal kidneys.

### Podocyte injury did not show time-dependent changes

**Figure 4:**
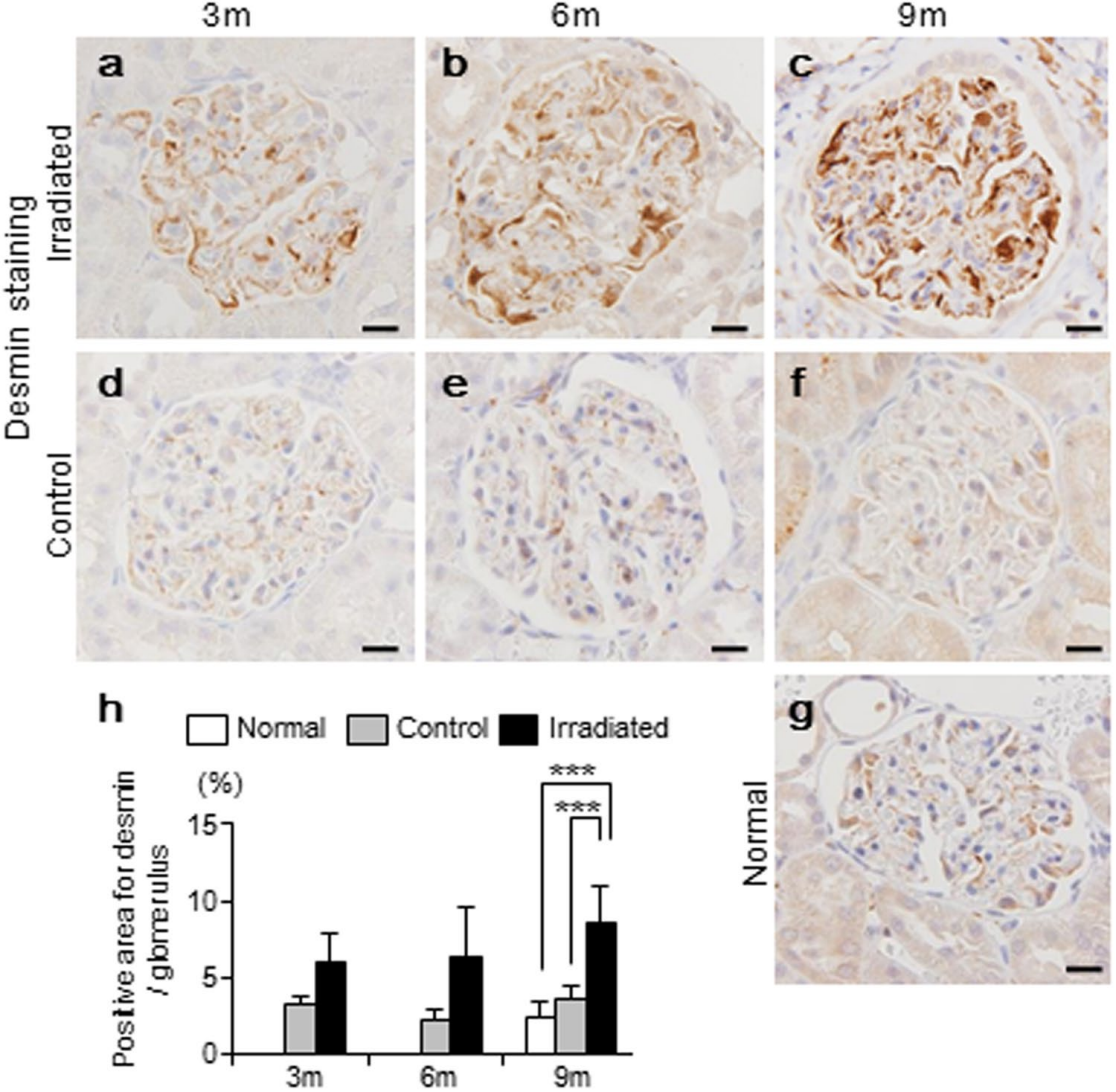
Quantification of podocyte injury by using desmin. (**a**–**g**, Bar, 20 µm) Representative images of immunohistochemistry for desmin (brown, a podocyte injury marker), counterstained for nuclei with hematoxylin (bleu). (**h**) Higher positive staining for desmin was found in irradiated kidneys than in control and normal kidneys at 9 months. However, irradiated kidney showed no time-dependent increase over 9 months. Values are expressed as mean ± SD. ***P < 0.001.

We next evaluated podocyte injury, by using staining for desmin, an intermediate filament protein (a marker of podocyte injury^28^) (Fig. 4a–g). At 9 months, irradiated kidneys showed a greater desmin-stained area compared to control and normal kidneys (irradiated, 8.6 ± 2.3% vs. control, 3.6 ± 0.8%, p < 0.001 and irradiated, 8.6 ± 2.3% vs. normal, 2.4 ± 1.0%, p < 0.001). There were no differences between control and normal kidneys. However, during 9 months, irradiated kidneys did not show time-dependent changes as seen as the decreased numbers of endothelial cells (Fig. 4h).

### Radiation-induced cellular senescence was seen in glomerular endothelial cells and podocytes

**Figure 5:**
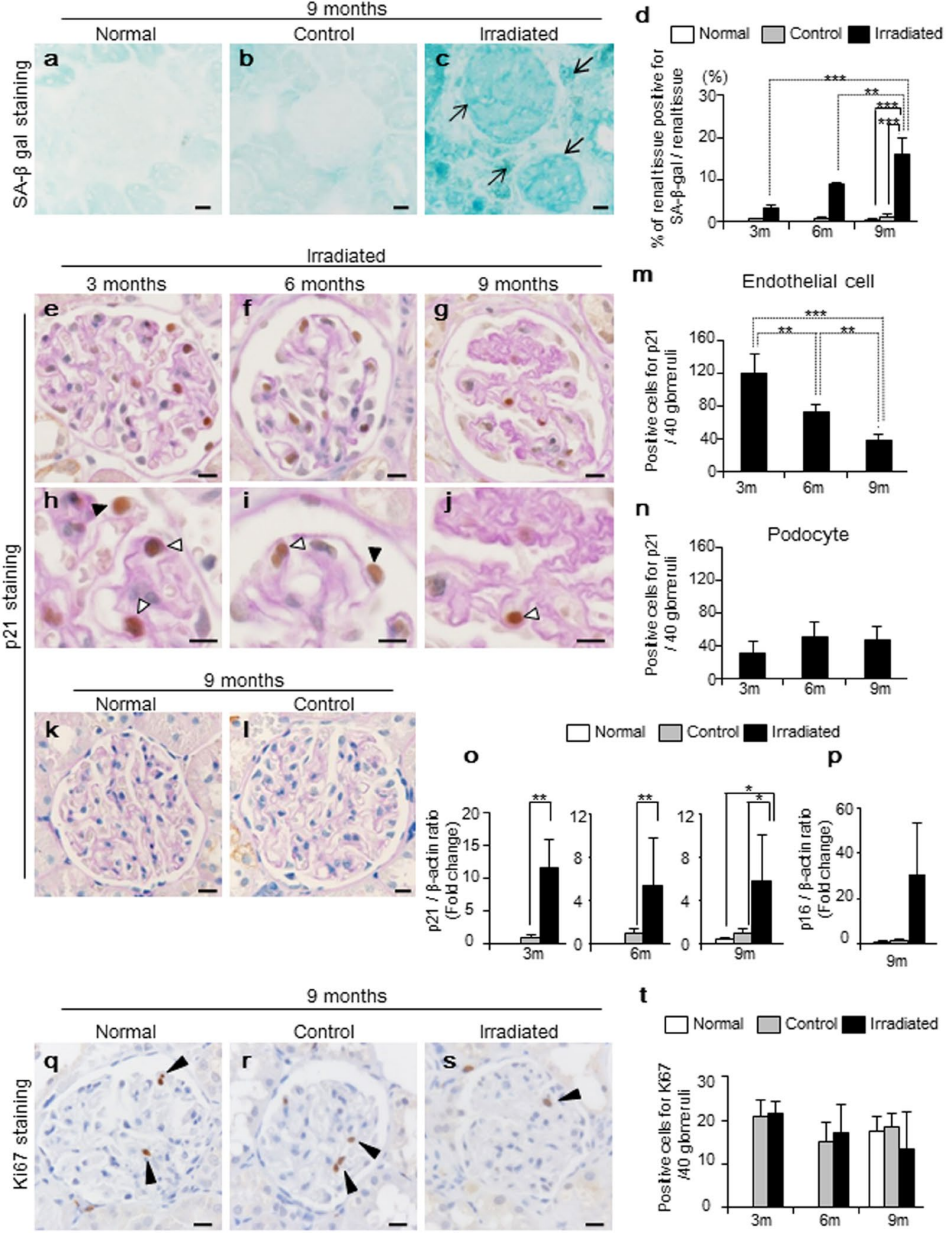
Assessments of radiation-induced cellular senescence by using SA-β-gal, p21, p16 and Ki-67. (**a**–**c**, Bar, 20 µm) Representative image of senescence-associated β-galactosidase (SA-β-gal) staining of kidney tissue at 9. Black arrows indicate positive staining for SA-β-gal. (**d**) Blue-stained areas were measured in 3 randomly-selected fields within each frozen section, and measurements presented as a percentage of the total area. Irradiated kidneys showed significant time-dependent increase in positive staining for SA-β-gal. (**e**–**l**, Bars, 20 µm) Representative images of p21 immunohistochemistry, counterstained with periodic acid-Schiff (PAS) staining. Both of endothelial cells and podocytes stained positive for p21 in irradiated kidneys at 3, 6, and 9 months (white and black arrows represent endothelial cells and podocytes, respectively). (**k**,**l**) Control and normal kidneys did not stain positive for p21 during 9 months. (**m**) Positive staining for p21 in endothelial cells was higher in earlier time, and gradually decreased in a time-dependent manner. (**n**) In contrast, positive staining for p21 in podocytes did not show time-dependent differences during 9 months. (**o**) Expression level of p21 mRNA obtained from isolated glomeruli. Irradiated kidneys showed higher expression levels of p21 mRNA at 3, 6, and 9 months compared to control and normal kidneys. (**p**) Additionally, irradiated kidneys showed relatively higher expression levels of p16 mRNA at 9 months compared to control and normal kidneys. (**q**–**s**, Bar, 20 µm) Representative images of immunohistochemistry for Ki-67 at 9 months. (black arrows represent positive staining for Ki-67). (**t**) There were no differences in positive staining for Ki-67 among normal, control, and irradiated kidneys over 9 months. Values are expressed as mean ± SD. *P < 0.05, **P < 0.01, and ***P < 0.001.

We evaluated whether and how radiation-induced cellular senescence occurred in the kidney. First, we examined SA-β-gal activity (Fig. 5a–c). The positively stained area continued to increase in irradiated kidneys at 3, 6, and 9 months in a time-dependent way, with means of 3.4 ± 0.6%, 8.8 ± 0.6%, and 15.9 ± 4.1%, respectively (Fig. 5d). At 9 months, irradiated kidneys showed higher positive stained area compared to control and normal kidneys (irradiated, 15.9 ± 4.1% vs. control, 1.2 ± 0.9%, p < 0.001 and irradiated, 15.9 ± 4.1% vs. normal, 0.64 ± 0.38%, p < 0.001) (Fig. 5d). There were no differences between control and normal kidneys.

As a second hallmark of cellular senescence, we examined cell cycle arrest by using p21 and p16 (Fig. 5e–p). Glomerular endothelial cells in irradiated kidneys showed higher number of positive staining for p21 in earlier time. Similar to the reduced number of CD31-positive glomerular endothelial cells, the cells with positive staining for p21 gradually decreased at 3, 6, and 9 months, with mean positive numbers of 121 ± 23, 73 ± 9, and 39 ± 6 (/40 glomeruli), respectively (Fig. 5m). In contrast, podocytes in irradiated kidneys showed certain positive staining, however there were no time-dependent differences at 3, 6, 9 months, with mean positive numbers of 32 ± 13, 52 ± 17, and 48 ± 16, respectively (/40 glomeruli)(Fig. 3n). We also quantified the expression levels of p21 and p16 mRNA obtained from isolated glomeruli (Fig. 5o,p). The expression levels of p21 mRNA were higher in irradiated kidneys than in the controls during 9 months, with the means being 11.6–fold higher at 3 months (p = 0.013), 5.4–fold higher at 6 months (p = 0.003), and 5.8–fold higher at 9 months (p = 0.040), respectively. At 9 months, irradiated kidneys also showed higher expression level of p21 mRNA compared to normal kidneys, with the means of 14.5–fold higher (p = 0.033).There were no differences between the control and normal kidneys at 9 months. Regarding p16, the irradiated kidneys showed relatively higher mRNA expression of p16 compared to control and normal kidneys at 9 months, although the differences were not significant (p = 0.082 and p = 0.078, respectively) (Fig. 5p). Up until 6 months, there were no significant differences between irradiated and control kidneys. The absence of DNA synthesis is another important feature of cell cycle arrest, then, Ki-67 (a marker of DNA synthesis) was evaluated (Fig. 5q–s). Notably, positive staining for Ki-67 was not different among irradiated, control and normal kidneys throughout 9 months (Fig. 5t). In addition, time-dependent changes were not seen in irradiated kidneys.

Radiation-induced cellular senescence in tubular cells was analyzed separately (See Supplementary Fig. 1)

### IL-6 was the major cytokine in senescence-associated secretory phenotype (SASP) *in vivo*

**Figure 6:**
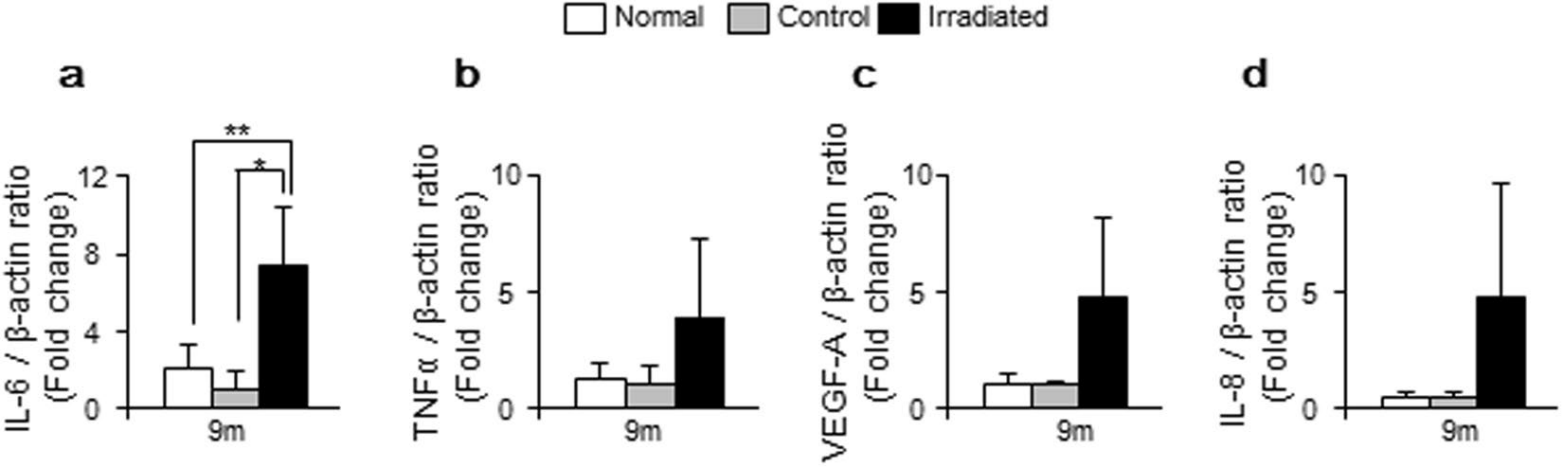
Analysis of senescence-associated secretory phenotype (SASP) by using quantitative real-time PCR. (**a**–**d**) SASP was assessed by examining the expression levels of IL-6, TNF-α, VEGF-A, and IL-8 mRNA at 9 months. (**a**) Irradiated kidneys showed a mean 7.4–fold and 3.6–fold higher expression level of IL-6 compared to control and normal kidneys, respectively. (**b**–**d**) Regarding TNF-α, VEGF-A, and IL-8, there were no statistical differences. Values are expressed as mean ± SD. *P < 0.05 and **P < 0.01.

We evaluated the expression levels of IL-6, TNF-α, VEGF-A, and IL-8 mRNA as SASP (Fig. 6a–d). Irradiated kidneys were found to have elevated expression level of IL-6 mRNA at 9 months compared to control and normal kidneys, with a mean 7.4–fold higher and 3.6–fold higher, respectively (p = 0.005 and 0.022, respectively) (Fig. 6a). There were also no differences between control and normal kidneys. Up until 6 months, there were no significant differences detected between irradiated and control kidneys. Regarding TNF-α, VEGF-A, and IL-8, irradiated kidneys had relatively higher mRNA expression levels compared to control and normal kidneys at 9 months in each case, but these differences were not statistically significant (Fig. 6b–d).

### DNA damage response pathway and subsequent cellular senescence process in glomerular endothelial cells *in vitro*

**Figure 7:**
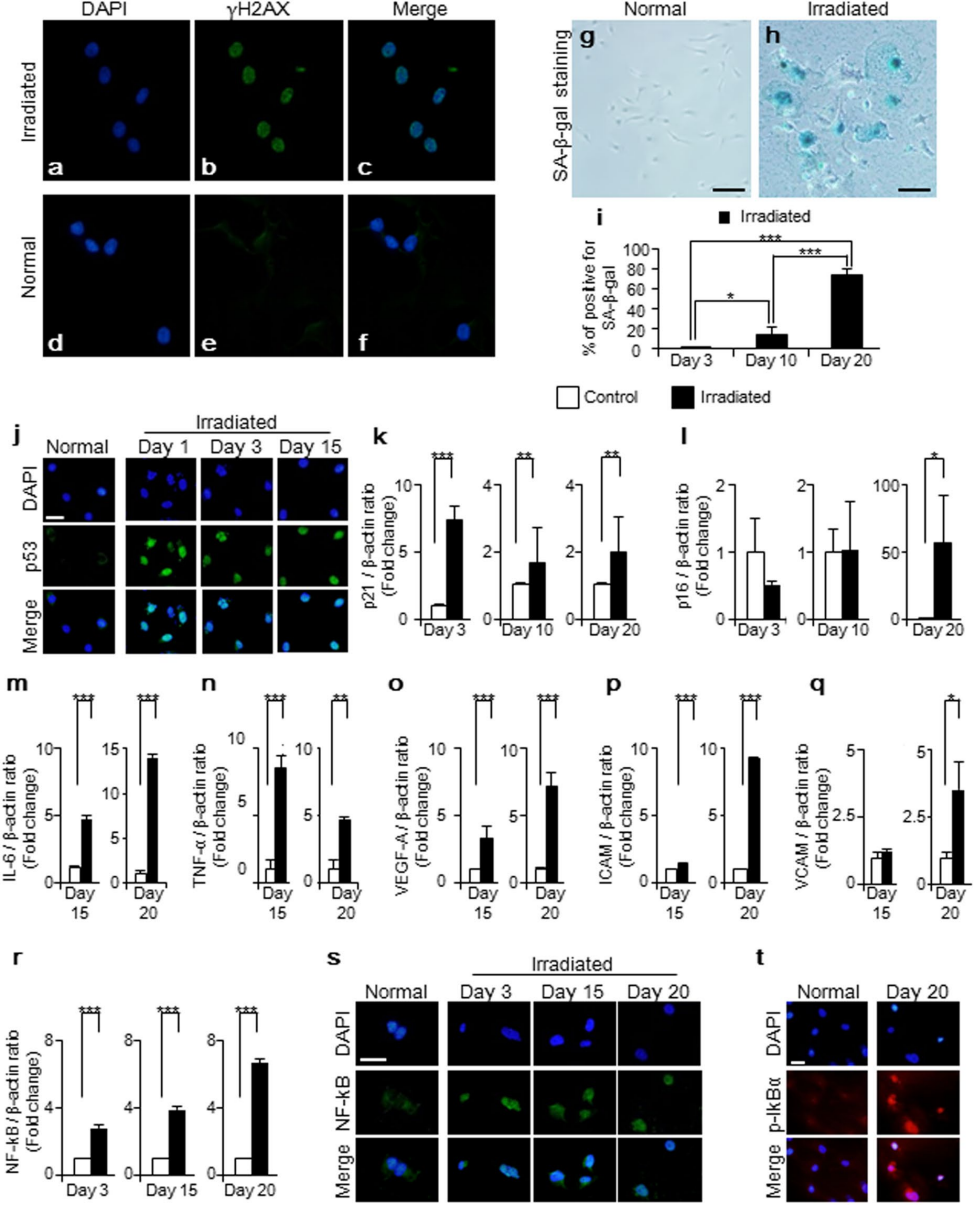
Radiation-induced DNA damage and subsequent cellular senescence *in-vitro* experiments with rat glomerular endothelial cells. (**a**–**f**) Radiation induced DNA double strand breaks (DSBs) in glomerular endothelial cells, shown as γH2AX (a DSBs marker). (green, γH2AX and bleu, nuclei). (**g**,**h**, Bar, 50 µm) Representative images of SA-β-gal staining in cultured glomerular endothelial cells at Days 0 and 20. (**i**) Irradiated glomerular endothelial cells displayed increase in positive staining for SA-β-gal in a time-dependent manner, at Days 3, 10 and 20. (**j**) Irradiated cells showed positive staining for p53 in the nucleus. (**k**) Irradiated cells sustained higher expression level of p21 mRNA compared to normal cells, at Days 3, 10, and 20. (**l**) Irradiated cells showed higher expression levels of p16 mRNA at Day 20. (**m**–**q**) SASP (IL-6, TNF-α, VEGF-A, ICAM and VCAM) was increased in irradiated glomerular endothelial cells at Days 15 and 20. (**r**–**t**, Bar 100 µm) Irradiated cells showed increase of expression level of mRNA. Additionally, they showed activation of NF-kB revealed by positive staining in nuclear localization and phosphorylated IκBα. Values are expressed as mean ± SD. *P < 0.05, **P < 0.01, and **P < 0.001.

We conducted *in vitro* experiments to confirm the characteristics of radiation-induced cellular senescence in glomerular endothelial cells. Radiation can induce DNA damage, as DNA double-strand breaks (DSBs). We first evaluated radiation-induced DSBs by using histone γH2AX, a surrogate marker of DSBs^29^. Irradiated cells showed positive staining for γH2AX 1 hour after radiation, which was not detected in normal cells (Fig. 7a–f). SA-β-gal activity was assessed *in vivo* experiments as well (Fig. 7g,h). The positively stained cells continued to increase after radiation in a time-dependent manner (Fig. 7i). Following DSBs, irradiated cells showed positive staining for p53 at Days 1, 3, and 15 (Fig. 7j). The expression levels of p21mRNA were up-regulated and sustained in irradiated cells, at Days 3, 10 and 20 (p < 0.01, p = 0.002, and p = 0.006, respectively) (Fig. 7k). Additionally, the expression levels of p16 mRNA were up-regulated in irradiated cells at Day 20 (p = 0.049) (Fig. 7l). SASP (including, IL-6, TNF-α, VEGF-A, ICAM, and VCAM) were upregulated at Days 15 and 20 (Fig. 7m–q). NF-kB signaling pathway was evaluated. In normal condition, NF-κB is inhibited by binding to IκB proteins, such as IκBα. Previous studies reported that DDR triggers phosphorylation of the inhibitory IκB proteins. The phosphorylated IκB proteins are released from NF-κB. Subsequently, NF-κB translocates to the nucleus and transactivates the expression of target genes. In our study, irradiated cells displayed gradual increase in expression level of NF-kB mRNA at Days 3, 15, and 20, with a mean 2.8–fold higher (p < 0.001), 3.9–fold higher (p < 0.001), and 6.7–fold higher (p < 0.001), respectively (Figure r). Irradiated cells showed positive staining for NF-κB, revealing nuclear localization. In contrast, normal cells showed positive staining for NF-κB, revealing cytoplasmic localization (Fig. 7s). In addition, irradiated cells showed positive staining for phosphorylated IκBα (p- IκBα) at Day 20 (Fig. 7t).

## Discussion

In the current study, we demonstrated radiation-induced cellular senescence in the glomerular endothelial cells and podocytes. Pathologically, glomerular endothelial cell injury was more severe, as implicated by the time-dependent increase in TMA, collapsing glomeruli, and decrease in the absolute number of endothelial cells. These pathological changes were accompanied by a decrease in glomerular endothelial cells that were positively stained for p21. We, therefore, suggested that radiation-induced cellular senescence was more likely to occur in the glomerular endothelial cells, and that they might be gradually damaged or lost. In combination with *in-vivo* and *in-vitro* analyses, we showed that SASP, mainly IL-6, was produced in senescent glomerular endothelial cells, which might follow the NF-kB signaling pathway. To our knowledge, this is the first study to demonstrate that radiation-induced cellular senescence, particularly derived from glomerular endothelial cells, may play an important role in the development of glomerular injury.

The first major outcome of this study was the verification of radiation-induced cellular senescence in the kidneys. Although identification of cellular senescence is challenging, commonly used markers, such as increased senescence-associated β-galactosidase (SA-β-gal) activity and irreversible cell cycle arrest, may be applied for its detection. Notably, irradiated kidneys showed time-dependent increase in SA-β-gal activity, thereby suggesting that cellular senescence might accumulate following the radiation. In the normal state, cells require the activation of cyclin dependent kinase (CDK) for further cell cycle progression^12^. Therefore, the detection of CDK inhibitors, such as p21 and p16, would be suggestive of cell cycle arrest^13^. In addition, the absence of DNA synthesis, implicated by the increase in Ki-67, BrdU (5-bromo-2-deoxyuridine), and Edu (5-Ethynyl-2′-deoxyuridine), also support cell cycle arrest^12,13,30^. In the current study, up regulation of p53, p21, and p16 and absence of Ki-67 indicated the irreversible cell cycle arrest following radiation.

As a second major finding, although cellular senescence was proven in glomerular endothelial cells and podocytes, the pathological findings displayed more severe glomerular endothelial cell injury, as evidenced by TMA and collapsing glomeruli. We considered glomerular endothelial cells to preferentially undergo radiation-induced senescence, resulting in cell damage and loss. In fact, several aging studies had focused on endothelial senescence induced by radiation^4,5,8,15,31^. These data reported that endothelial cells were particularly susceptible to radiation, and that radiation-induced endothelial senescence was an obligatory factor in the development of diseases, including cardiovascular and neurovascular diseases^4,8^.

In the current study, the absolute number of endothelial cells gradually decreased. In addition, cells with positive staining for p21 were more in number than endothelial cells at an earlier time. These positive numbers showed a gradual time-dependent decrease. Concomitant with this decrease, prevalence of TMA and collapsing glomeruli increased in a time-dependent manner. In particular, collapsing glomeruli, in which no endothelial cell was preserved, accounted for about 50% of glomeruli at nine months. These parallel changes led us to presume that radiation-induced senescence preferentially occurred in the glomerular endothelial cells, eventually leading to cell damage and loss. This loss could be explained by the loss of p21-positive senescent glomerular endothelial cells, along with impaired angiogenic capacity. Angiogenic capacity was reported to be impaired in radiation-induced senescent endothelial cells, with reduced capillary numbers^14,26,32^. Together, the results suggested that senescent glomerular endothelial cells might decrease their ability of angiogenesis to replace the damaged cells. In collapsing glomeruli, almost no capillary lumen was preserved, due to which glomerular filtration rate (GFR) could be remarkably reduced. We suggested that reduced GFR might contribute to chronic renal failure, as shown in elevated BUN at nine months.

Compared to endothelial cells, absolute number of positive p21-staining in podocytes was less. In addition, these positive numbers did not significantly change during the nine months. The desmin-positive area also did not change significantly during the nine months. Although podocyte foot process effacement was observed in electron microscopic analysis, podocyte detachment was apparently not seen. Therefore, we suggested that podocytes may be relatively resilient to the radiation-induced cellular senescence. In fact, it had earlier been reported that the type of senescent cells may vary according to the individual stress factors involved^33^.

Recently, there has been growing interest in the possible role played by cellular senescence in the development of glomerular diseases and CKD^12,33^. For example, IgA nephropathy, membranous nephropathy, minimal change disease, and focal segmental glomerular sclerosis showed increased expression of senescent markers, such as SA-β-gal activity, p21, and p16, thereby suggesting a potential relationship between cellular senescence and glomerular disease progression^34,35^. Diabetic nephropathy, the most common cause of CKD, was also reported to be associated with cellular senescence, in which hyperglycemia was the major stress factor that induced cellular senescence. Therapy-induced senescence is another serious concern in CKD. Kidney transplantation is a well-known therapy-induced senescence, in which ischemic reperfusion injury, acute rejection, hypertension, and allograft rejection all acted as stress factors that induced cellular senescence^36,37^. Herein, based on our experiments, we propose that radiation-induced cellular senescence may be an important risk factor in the progression of glomerular injury and subsequent chronic renal failure. It may be crucial for clinicians to remember the long-term adverse effects of radiation related to cellular senescence, and it may be reasonable to avoid unnecessary or excessive use of radiation.

Based on *in-vivo* analysis, we conducted *in-vitro* experiments with cultured glomerular endothelial cells from rats. Radiation is known to cause DNA double-strand breaks (DSBs), which are the most life-threatening form of genomic insult^38,39^. To avoid such genomic instability, cells have a preventive network system, called the DNA damage response (DDR). The DDR pathway recruits several components to the DSBs, finally leading to cellular senescence. Importantly, when cells become senescent, they acquire many changes in gene expression, namely, senescence-associated secretory phenotype (SASP)^33,40^. SASP includes a variety of cytokines, chemokines, and growth factors, such as, IL-6, IL-8, IL-1, TNF-α, MCP-1, PAI-1, VEGF, ICAM, and VCAM-1^14,41–43^. Recently, NF-kB signaling pathway was demonstrated as the major inducer of SASP that could be triggered by DDR^44,45^. In addition to DDR, SASP itself, such as IL-1 and TNF-α, were also known to activate NF-kB signaling pathway^45^. The secreted IL-6, IL-1, IL-8, and VEGF were reported to further amplify cellular senescence in the neighboring cells, as a paracrine effect^46^.

In the current study, we observed early radiation-induced DNA DSBs, an initial process of DDR, with subsequent up regulation of NF-kB signaling. At a later period, higher NF-kB and SASP (IL-6, TNF-α, VEGF-A, ICAM, and VCAM) expression levels were observed. We speculated that NF-kB signaling pathway might play an important role in SASP, which would contribute to further increase in NF-kB signaling at a later period. In combination with *in-vivo* analysis, IL-6 may be the most promising SASP in radiation-induced glomerular endothelial cellular senescence.

In conclusion, our current study provided a new insight into the contribution of radiation-induced cellular senescence, in particular for glomerular endothelial cells, to the development of glomerular TMA and collapsing glomeruli with renal dysfunction. These findings may attract further attention for a possible risk of CKD in relation to cellular senescence. In the future, further strategies that will be explored include the use of geroprotectors or senolysis to prevent or ameliorate CKD relating to cellular senescence.

## Materials and Methods

### Study Approval

All the animal experiments described in this study were approved by the Animal Experiments Ethical Review Committee of Nippon Medical School (Tokyo, Japan). The approved number is 30–312. All the methods were performed in accordance with approved guidelines and regulation for the care and use of laboratory animals of Nippon Medical School.

### Animals

Experiments were performed in 7- to 8- week-old male Dark Agouti rats with body weights of 150–190 g, which were obtained from Sankyo Laboratory Service Corporation (Tokyo, Japan). Twelve rats received radiation in a randomly selected unilateral kidney (irradiated kidney). The other kidney was shielded with lead (control kidney). The remainder of the body including the bone marrow was also shielded. A single dose of 18 Gy was delivered at a dose rate of 1.34 Gy/minute to the selected kidney posteriorly and anteriorly with 0.5 mm Al + 0.1 mm Cu filtration using MBR-1520R-3 (Hitachi Ltd, Tokyo, Japan). The design of animal model was consistent with the previous studies, in which unilateral radiation showed clinical kidney toxicity^24,27^. Radiation dose was confirmed by preliminary experiments (data not shown). During radiation, the rats were immobilized by anesthetization with isoflurane and medetomidine (0.15 mg/kg). Five rats received sham radiation (normal kidney). At 3, 6, and 9 months after radiation, rats were anesthetized for blood sample collection, subsequently they were euthanized and renal tissue was collected (irradiated rats, at 3 months, n = 3; at 6 months, n = 3; and at 9 months, n = 6; and normal rats, at 9 months, n = 5).

As biological indicators, the body weight and blood pressure (BP) were measured pre and at 3, 6, and 9 months after radiation/sham radiation. Urine samples were collected over a 24-hour period pre and at 3, 6, and 9 months after radiation/sham radiation. Renal function was assessed by proteinuria, the level of blood urea nitrogen (BUN), and the level of hemoglobin (Hb), pre and at 3, 6, and 9 months after radiation/sham radiation. The concentration of proteinuria and the level of BUN were measured by a commercial company (Oriental Yeast Co, Shiga, Japan).

### Light and Electron microscopy

Periodic acid-silver methenamine (PASM) staining was performed for light microscopic analysis. Kidneys were fixed with 10% buffered formalin and embedded in paraffin. Paraffin sections of 1.5 µm thickness were processed. De-paraffinized sections were treated with 1% periodic acid for 15 min. Sections were washed in distilled water and incubated with 0.5% thiosemicarbazide for 5 min. Sections were incubated in methenamine silver solution, containing 3% hexamethylenetetramine, 5% silver nitrate, and 5% sodium tetraborate in distilled water, at 60 °C for 45 min. Sections were washed and incubated in 0.25% gold chloride for 10 min, in 2% oxalic acid formalin for 1 min, and in fixing solution for 10 min. Sections were washed and counter-stained with hematoxylin and eosin. Sections were examined with OLYMPUS BX05 (Olympus Corporation, Tokyo, Japan).

For electron microscopic analysis, kidney tissues were fixed with 2.5% glutaraldehyde in phosphate buffer (pH 7.4), post-fixed with 1% osmium tetroxide, dehydrated, and embedded in Epon 812 (Okenshoji, Tokyo, Japan). Ultrathin sections were stained with uranyl acetate, lead citrate, and oolong tea extract, then examined with an electron microscope using Hitachi H 7650 (Hitachi, Yokohama, Japan).

### SA-β-gal staining

The staining procedure was based on a reported protocol^40,47^. Briefly, the kidney tissues were embedded in OCT compound (4583, Sakura Finetek, CA, USA) and immediately cut into 8 µm sections. Tissues were fixed in 4% paraformaldehyde (PFA) at 4 °C for 1 hour. Cultured rat glomerular endothelial cells were fixed with 2% PFA at room temperature for 5 minutes. Fixed tissues or cultured cells were stained with solution containing 40 mmol/L citric acid-sodium phosphate (pH 6.0), 150 mmol/L NaCl, 2 mmol/L MgCl_2_, 5 mmol/L potassium ferricyanide, 5 mmol/L potassium ferrocyanide, and fresh 1 mg/mL 5-Bromo-4-chloro-3-indolyl-β-D-galactoside (X-gal; 05644-14, Nacalai tesque, Tokyo, Japan) at 37 °C (without CO_2_) overnight.

### Real-time quantitative polymerase chain reaction (RT-qPCR)

Total RNA was extracted from isolated glomeruli and cultured rat glomerular endothelial cells using ISOGEN (Nippon Gene, Tokyo, Japan) according to the manufacturer’s protocol. The mRNA concentration was determined using a Nano Drop ND 1000 V 3.8.1 Spectrophotometer (Nano Drop Technologies, DE, USA). Total RNA was reverse-transcribed to cDNA using the High Capacity cDNA Reverse Transcription kit (Applied Biosystems, CA, USA). Quantitative real-time polymerase chain reaction was performed using THUNDERBIRD SYBR qPCR Mix (TOYOBO, Osaka, Japan) in triplicate. All measured values were normalized to β-actin gene expression and analyzed using the ⊿⊿C_T_ method. Fold change values (calculated by the formula 2^−⊿⊿CT^) were used as final expression data. Primer sequences used are: p21, 5′-CCG TGG ACA GTG AGC AGT TG-3′ (forward) and 5′-CGT CTC AGT GGC GAA GTC AA-3′ (reverse); p16, 5′-TGC AGA TAG ACT AGC CAG GGC A-3′ (forward) and 5′-CTT CCA GCA GTG CCC GCA-3′ (reverse), IL-6, 5′-GTC AAC TCC ATC TGC CCT TCA G-3′ (forward) and 5′-GGC AGT GGC TGT CAA CAA CAT-3′ (reverse); TNF-α, 5′-AAA TGG GCT CCC TCT CAT CAG TTC-3′ (forward) and 5′-TCT GCT TGG TGG TTT GCT ACG AC-3′ (reverse), VEGF-A, 5′-TGT GCG GGC TGC TGC AAT GA-8′ (forward) and 5′-TGT GCT GGC TTT GGT GAG GTT TG-8′ (reverse), IL-8, 5′-CCC CCA TGG TTC AGA AGA TTG-3′ (forward) and 5′-TTG TCA GAA GCC AGC GTT CAC-3′ (reverse), NF-kB, 5′-GGC AGC ACT CCT TAT CAA-3′ (forward) and 5′-GGTGTC GTC CCA TCG TAG-3′(reverse), ICAM, 5′-CGT GGC GTC CAT TTA CAC CT-3′ (reverse) and 5′-TTA GGG CCT CCT CCT GAG C-3′ (reverse), and VCAM 5′-GCG AAG GAA ACT GGA GAA GAC A-3′ (forward) and 5′-ACA CAT TAG GGA CCG TGC AGT T-3′ (reverse), and β-actin, 5′-ACC ACC ATG TAC CCA GGC ATT-3′ (forward) and 5′-CCA CAC AGA GTA CTT GCG CTC A-3′ (reverse).

### Immunohistochemistry

Paraffin sections with thicknesses of 3 µm were processed for immunohistochemistry. For labeling with primary antibodies, the sections were deparaffinized and treated for 30 min with 0.3% H_2_O_2_ in methanol. All the sections were heated in an autoclave for 20 min in a citrate buffer (pH 6.0), then incubated with the primary antibodies overnight. The primary antibodies and conditions are as follow: anti-p21 (1:25, mouse monoclonal, NBP2-29463, Novus Biologicals, CO, USA); anti-Ki-67 (1:800, mouse monoclonal, M7248, Dako, CA, USA), anti-CD31 (1:60, goat monoclonal, AF3628, R&D Systems, MN, USA), and anti-desmin (1:80, mouse monoclonal, M0760, Dako, CA, USA). Sections were stained with biotinylated anti-mouse IgG or anti-goat IgG (Dako, CA, USA) and visualized using H_2_O_2_-containing diaminobenzidine buffer.

### Immunocytochemistry

Immunostaining of γH2AX, p53, and NF-kB was followed by instructed protocol. In brief, cultured glomerular endothelial cells were fixed in 4% PFA for 15 minutes, permeabilized with 0.5% Triton X-100 in PBS for 15 minutes, and blocked with 3% bovine serum albumin in PBS for 60 minutes. All the procedures were performed at room temperature. Cells were then incubated with primary antibody, anti-γH2AX (1:500, rabbit monoclonal, 9718, Cell Signaling Technology, MA, USA), anti-p53 (1:50, rabbit polyclonal, 10442-1-AP, Proteintech, IL, USA), anti-NF-kB (1:100, rabbit polyclonal, PA5-16545, Thermo Fisher, IL, USA), and anti-p-IκBα (1:50, mouse monoclonal, santa cruz, sc-8404, TX, USA) at 4 °C overnight. Cells were incubated with Alexa Fluor Plus 488 goat anti-rabbit IgG secondary antibody (1:500, A32731, Thermo Fisher Scientific, Tokyo, Japan), at room temperature for 1 hour. Nuclei were counterstained with DAPI (H-1200, Vector Laboratories, CA, USA).

### Cell experiments

Rat glomerular endothelial cells and medium containing several growth factors and supplements were purchased from Cell Biologics (RA-6014G and M1266-lit, Cell Biologics, Chicago, USA). The cells (passages 11–15) were cultured at 37 °C in a humidified incubator containing 5% CO_2_. The cells (8 × 10^4^) were seeded in a 6-cm dish. The day after seeding, a single dose of 20 Gy was delivered at a dose rate of 4.96 Gy/min using OM-100R OHMiC SOFT X-RAY SYSTEM (Omic Corporation, Shiga, Japan). According to the previous study, the irradiated cells were not passaged, but the medium was partially changed on Days 3, 8, and 13^14^.

### Quantification of pathological findings

For evaluation of pathological findings, forty randomly selected glomeruli (200x) were examined and classified into thrombotic microangiopathy (TMA) and collapsing glomeruli. For evaluation of positive staining for p21, Ki-67, and CD31were examined (200x). We counted the number of positively-stained cells in each staining. For evaluation of desmin, forty randomly selected glomeruli (200x) were examined using color image analysis software (WinROOF; Mitani Co., Tokyo, Japan). Positive binary staining threshold was applied to all the images automatically to detect the percentage of positive area. For evaluation of positive staining for SA-β-gal *in-vivo* experiments, three randomly selected fields (200x) were evaluated. Similar to the evaluation of desmin, color image analysis software was applied to detect positive staining for SA-β-gal (WinROOF; Mitani Co., Tokyo, Japan). *In-vitro* experiments, three randomly selected areas (100x) were evaluated. In each area, 100 randomly selected glomerular endothelial cells were evaluated and counted as the number of cells with positive staining for SA-β-gal.

### Statistical analysis

All data are represented as means ± SD. The Student *t*-test and analysis of variance model (ANOVA) followed by Tukey test were applied for comparisons of continuous data between two and three groups, respectively.

A p value < 0.05 was considered statistically significant. We conducted all statistical analyses using the R software package (version 3.3.3, R Development Core Team, https://www.r-project.org/).

## Electronic supplementary material

Supplementary Information

## Data Availability

The data generated and/or analyzed in the current study are not publicly available; however, they are available from the corresponding author on reasonable request.

## References

[1] Childs BG, Durik M, Baker DJ, Van Deursen JM (2015). Cellular senescence in aging and age-related disease: from mechanisms to therapy. Nat Med..

[2] Blagosklonny MV (2012). Prospective treatment of age-related diseases by slowing down aging. Am J Pathol..

[3] O’sullivan ED, Hughes J, Ferenbach DA (2017). Renal Aging: Causes and Consequences. J Am Soc Nephrol.

[4] Wang Y, Boerma M, Zhou D (2016). Ionizing Radiation-Induced Endothelial Cell Senescence and Cardiovascular Diseases. Radiat Res..

[5] Tapio S (2016). Pathology and biology of radiation-induced cardiac disease. J Radiat Res..

[6] Citrin DE (2013). Role of type II pneumocyte senescence in radiation-induced lung fibrosis. J Natl Cancer Inst..

[7] Schafer MJ (2017). Cellular senescence mediates fibrotic pulmonary disease. Nat Commun..

[8] Mcrobb LS (2017). Ionizing radiation reduces ADAM10 expression in brain microvascular endothelial cells undergoing stress-induced senescence. Aging (Albany NY)..

[9] Cheng Z, Zheng YZ, Li YQ, Wong CS (2017). Cellular Senescence in Mouse Hippocampus After Irradiation and the Role of p53 and p21. J Neuropathol Exp Neurol..

[10] Mccart, E. A. *et al*. Accelerated senescence in skin in a murine model of radiation-induced multi-organ injury. *J Radiat Res* 1–11 (2017). 10.1093/jrr/rrx008 PMC5737212 28340212

[11] Wang Y, Schulte BA, Larue AC, Ogawa M, Zhou D (2006). Total body irradiation selectively induces murine hematopoietic stem cell senescence. Blood..

[12] Sturmlechner I, Durik M, Sieben CJ, Baker DJ, Van Deursen JM (2017). Cellular senescence in renal ageing and disease. Nat Rev Nephrol..

[13] Bernardes De Jesus B, Blasco MA (2012). Assessing cell and organ senescence biomarkers. Circ Res..

[14] Ungvari Z (2013). Ionizing radiation promotes the acquisition of a senescence-associated secretory phenotype and impairs angiogenic capacity in cerebromicrovascular endothelial cells: role of increased DNA damage and decreased DNA repair capacity in microvascular radiosensitivity. J Gerontol A Biol Sci Med Sci..

[15] Lafargue Audrey, Degorre Charlotte, Corre Isabelle, Alves-Guerra Marie-Clotilde, Gaugler Marie-Hélène, Vallette François, Pecqueur Claire, Paris François (2017). Ionizing radiation induces long-term senescence in endothelial cells through mitochondrial respiratory complex II dysfunction and superoxide generation. Free Radical Biology and Medicine.

[16] Kitada K (2014). Hyperglycemia causes cellular senescence via a SGLT2- and p21-dependent pathway in proximal tubules in the early stage of diabetic nephropathy. J Diabetes Complications..

[17] Bhayadia R, Schmidt BM, Melk A, Homme M (2016). Senescence-Induced Oxidative Stress Causes Endothelial Dysfunction. J Gerontol A Biol Sci Med Sci..

[18] Zuelzer WW, Palmer HD, Newton WA (1950). Unusual glomerulonephritis in young children probably radiation nephritis. Am J Pathol..

[19] Emami B (1991). Tolerance of normal tissue to therapeutic irradiation. Int J Radiat Oncol Biol Phys..

[20] Domagk G (1927). Rontgentrahlenschadigungen der nierebein menschen. Med Klin..

[21] Cohen EP, Robbins ME (2003). Radiation nephropathy. Semin Nephrol..

[22] Dawson, L. A. *et al*. Radiation-associated kidney injury. Int J Radiat Oncol Biol Phys 5.133(20180430). **76**, S108–115 (2010). 10.1016/j.ijrobp.2009.02.089 20171504

[23] Rosen S, Swerdlow MA, Muehrcke RC, Pirani CL (1964). Radiation nephritis; light and electron microscopic observations. Am J Clin Pathol..

[24] Madrazo A, Suzuki Y, Churg J (1970). Radiation nephritis. II. Chronic changes after high doses of radiation. Am J Pathol..

[25] Jennette, J. C., D’Agati, V. D., Olson, J. L. & Silva, F. G. Heptinstall’s Pathology of the Kidney, 7th Edition (2014).

[26] Schultz-Hector S, Balz K (1994). Radiation-induced loss of endothelial alkaline phosphatase activity and development of myocardial degeneration. An ultrastructural study. Lab Invest..

[27] Welz S (2007). Renal toxicity of adjuvant chemoradiotherapy with cisplatin in gastric cancer. Int J Radiat Oncol Biol Phys..

[28] Herrmann A, Tozzo E, Funk J (2012). Semi-automated quantitative image analysis of podocyte desmin immunoreactivity as a sensitive marker for acute glomerular damage in the rat puromycin aminonucleoside nephrosis (PAN) model. Exp Toxicol Pathol..

[29] Kuo LJ, Yang LX (2008). Gamma-H2AX - a novel biomarker for DNA double-strand breaks. In Vivo..

[30] Mannava S (2013). Depletion of deoxyribonucleotide pools is an endogenous source of DNA damage in cells undergoing oncogene-induced senescence. Am J Pathol..

[31] Azimzadeh O (2015). Integrative proteomics and targeted transcriptomics analyses in cardiac endothelial cells unravel mechanisms of long-term radiation-induced vascular dysfunction. J Proteome Res..

[32] Igarashi K, Sakimoto I, Kataoka K, Ohta K, Miura M (2007). Radiation-induced senescence-like phenotype in proliferating and plateau-phase vascular endothelial cells. Exp Cell Res..

[33] Valentijn FA, Falke LL, Nguyen TQ, Goldschmeding R (2018). Cellular senescence in the aging and diseased kidney. J Cell Commun Signal..

[34] Sis B (2007). Accelerated expression of senescence associated cell cycle inhibitor p16INK4A in kidneys with glomerular disease. Kidney Int..

[35] Liu J (2012). Accelerated senescence of renal tubular epithelial cells is associated with disease progression of patients with immunoglobulin A (IgA) nephropathy. Transl Res..

[36] Schmitt R, Melk A (2012). New insights on molecular mechanisms of renal aging. Am J Transplant..

[37] Chkhotua AB (2003). Increased expression ofp16(INK4a) and p27(Kip1) cyclin-dependent kinase inhibitor genes in aging human kidney and chronic allograft nephropathy. Am J Kidney Dis..

[38] Van Attikum H, Gasser SM (2009). Crosstalk between histone modifications during the DNA damage response. Trends Cell Biol..

[39] Li M, You L, Xue J, Lu Y (2018). Ionizing Radiation-Induced Cellular Senescence in Normal, Non-transformed Cells and the Involved DNA Damage Response: A Mini Review. Front Pharmacol..

[40] Dimri GP (1995). A biomarker that identifies senescent human cells in culture and in aging skin *in vivo*. Proc Natl Acad Sci USA.

[41] Rodier F (2009). Persistent DNA damage signalling triggers senescence-associated inflammatory cytokine secretion. Nat Cell Biol..

[42] Coppe JP, Desprez PY, Krtolica A, Campisi J (2010). The senescence-associated secretory phenotype: the dark side of tumor suppression. Annu Rev Pathol..

[43] Prattichizzo F (2016). “Inflammaging” as a Druggable Target: A Senescence-Associated Secretory Phenotype-Centered View of Type 2 Diabetes. Oxid Med Cell Longev..

[44] Meyer P (2017). A model of the onset of the senescence associated secretory phenotype after DNA damage induced senescence. PLoS Comput Biol..

[45] Salminen A, Kauppinen A, Kaarniranta K (2012). Emerging role of NF-kappaB signaling in the induction of senescence-associated secretory phenotype (SASP). Cell Signal..

[46] Shakeri, H., Lemmens, K., Gevaert, A. B., De Meyer, G. R. Y. & Segers, V. Cellular senescence links aging and diabetes in cardiovascular disease. *Am J Physiol Heart Circ Physiol* (2018). 10.1152/ajpheart.00287.2018 29750567

[47] Debacq-Chainiaux F, Erusalimsky JD, Campisi J, Toussaint O (2009). Protocols to detect senescence-associated beta-galactosidase (SA-betagal) activity, a biomarker of senescent cells in culture and *in vivo*. Nat Protoc..

