# Supplementary material for: Radiation-induced premature cellular senescence involved in glomerular diseases in rats
Source: Sci Rep. 2018 Nov 14;8:16812. doi: 10.1038/s41598-018-34893-8 (PMC6235850; doi:10.1038/s41598-018-34893-8)
Supplement: Supplementary file 1 — Supplementary Information [file 41598_2018_34893_MOESM1_ESM.pdf]

Radiation-induced premature cellular senescence involved in glomerular diseases in rats.

*Sae Aratani, M.D.[1, 2], Masako Tagawa, M.D.[2], Shinya Nagasaka, PhD [2],

Yukinao Sakai, M.D., PhD [1], Akira Shimizu M.D., PhD [2], Shuichi Tsuruoka M.D., PhD [1]

[1] Department of Nephrology, Graduate School of Medicine, Nippon Medical School, Tokyo, Japan

[2] Department of Analytic Human Pathology, Graduate School of Medicine, Nippon Medical School, Tokyo, Japan

Correspondence:

Sae Aratani, M.D.

Department of Nephrology, Graduate School of Medicine, Nippon Medical School, Tokyo, Japan

Address: 1-1-5 Sendagi, Bunkyo-ku, Tokyo 113-8603, Japan

Phone: (+81)-3-3822-2131 (ext. 26496) Fax: (+81)-3-3822-4865

Email: sae-aratani@nms.ac.jp

Figure Legends

Supplementary Figure 1 Assessments of radiation-induced cellular senescence in tubular cells by using SA-β-gal, p21 and Ki-67. Tubular cells in irradiated kidneys showed several senescent markers including positive staining for SA-β-gal and p21 and the absence of the proliferation marker Ki-67. (a-c, Bars, 100 μm) Representative image of senescence-associated β-galactosidase (SA-β-gal) staining of kidney tissue at 9 months after irradiation. Black arrows indicate positive staining for SA-β-gal. (d-f, Bars, 50 μm) Representative images of p21 immunohistochemistry, counterstained with periodic acid-Schiff (PAS) staining. Positive staining for p21 was detected only in irradiated tubular cells. (g-i, Bars, 50 μm). Representative images of immunohistochemistry for Ki-67. There was no increase in positive staining for Ki-67 in irradiated tubular cells.

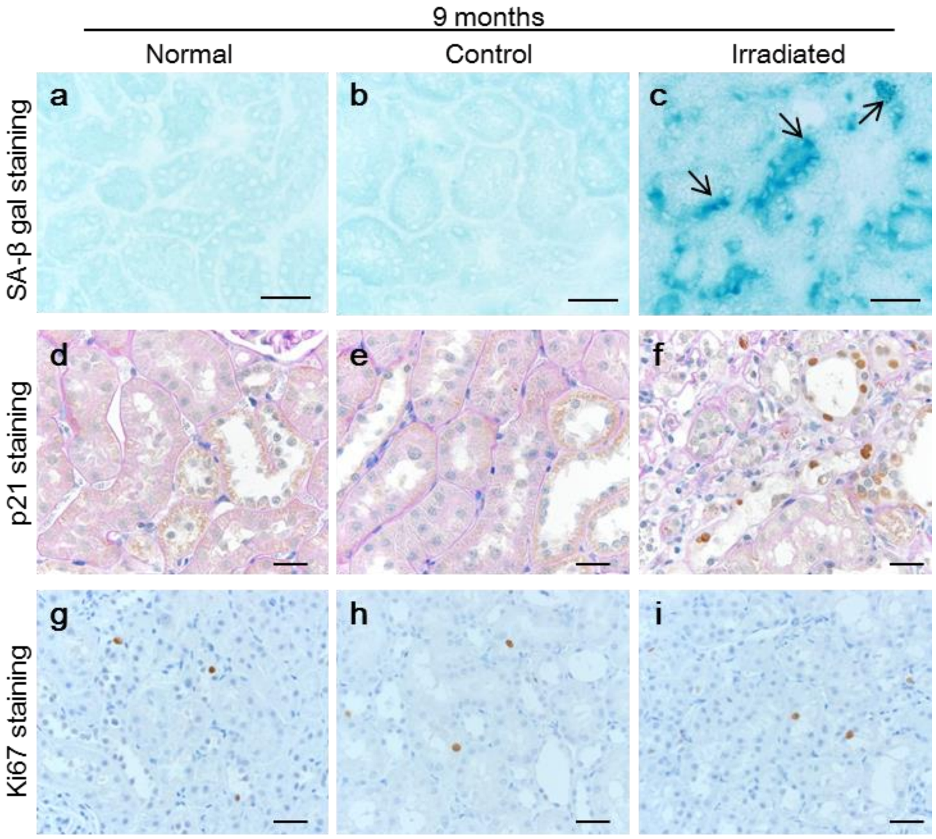
9 months
Normal
Control
Irradiated
SA-β gal staining
p21 staining
Ki67 staining
a
b
c
d
e
f
g
h
i
